# Exploring common biomarkers of ischemic stroke and obstructive sleep apnea through bioinformatics analysis

**DOI:** 10.1371/journal.pone.0312013

**Published:** 2024-10-30

**Authors:** Zhe Wu, Yutong Qian, Yaxin Shang, Yu Zhang, Meilin Wang, Mingyuan Jiao

**Affiliations:** 1 Rehabilitation Department, The Second Affiliated Hospital Zhejiang University School of Medicine, Hangzhou, P.R. China; 2 School of Acupuncture-Moxibustion and Tuina, Shanghai University of Chinese Medicine, Shanghai, P.R. China; 3 First Clinical Medical College, Heilongjiang University of Chinese Medicine, Harbin, P.R. China; 4 Department of Integrated Traditional Chinese and Western Medicine in Gynecology, Shanghai Jiading Maternal Child Health Hospital, Shanghai, P.R. China; 5 Department of Orthopedic and Spinal Rehabilitation, Ningbo Rehabilitation Hospital, Ningbo, P.R. China; 6 Research and Teaching Department, Jinhua Maternal Child Health Hospital, Jinhua, P.R. China; Cairo University Kasr Alainy Faculty of Medicine, EGYPT

## Abstract

**Background:**

Clinical observations have shown that many patients with ischemic stroke (IS) have a history of obstructive sleep apnea (OSA) both before and after the stroke’s onset, suggesting potential underlying connections and shared comorbid mechanisms between the two conditions. The aim of this study is to identify the genetic characteristics of OSA patients who develop IS and to establish a reliable disease diagnostic model to assess the risk of IS in OSA patients.

**Methods:**

We selected IS and OSA datasets from the Gene Expression Omnibus (GEO) database as training sets. Core genes were identified using the Limma package, Weighted Gene Co-expression Network Analysis (WGCNA), and machine learning algorithms. Gene Set Variation Analysis (GSVA) was conducted for pathway enrichment analysis, while single-sample gene set enrichment analysis (ssGSEA) was employed for immune infiltration analysis. Finally, a diagnostic model was developed using Least Absolute Shrinkage and Selection Operator (LASSO) regression, with its diagnostic efficacy validated using receiver operating characteristic (ROC) curves across two independent validation sets.

**Results:**

The results revealed that differential analysis and machine learning identified two common genes, *TM9SF2* and *CCL8*, shared between IS and OSA. Additionally, seven signaling pathways were found to be commonly upregulated in both conditions. Immune infiltration analysis demonstrated a significant decrease in monocyte levels, with *TM9SF2* showing a negative correlation and *CCL8* showing a positive correlation with monocytes. The diagnostic model we developed exhibited excellent predictive value in the validation set.

**Conclusions:**

In summary, two immune-related core genes, *TM9SF2* and *CCL8*, were identified as common to both IS and OSA. The diagnostic model developed based on these genes may be used to predict the risk of IS in OSA patients.

## 1. Introduction

Stroke is an acute cerebrovascular disease and the second leading cause of death worldwide. Ischemic stroke (IS), which accounts for the majority of stroke cases, occurs when blood flow to the brain is reduced or interrupted due to cerebral vascular obstruction. From 1990 to 2019, the global incidence of stroke increased by 70%, and stroke-related deaths rose by 22%. IS accounts for 62.4% of all strokes, posing a significant threat to human life and health [1]. Therefore, it is essential to understand and prevent the occurrence of IS. Obstructive sleep apnea (OSA) is an independent risk factor for IS, characterized by repeated episodes of breathing cessation or reduced breathing during sleep, typically caused by partial or complete obstruction of the throat tissues. Clinical trials have shown that the incidence of IS in OSA patients is 25.4%, compared to only 8.2% in non-OSA individuals, a statistically significant difference between the two groups [2]. OSA is also a common complication of IS, with a reported incidence of 21%, and as many as 79% of IS patients are affected by OSA [3]. Intracranial carotid artery calcification (ICAC), a marker of cardiovascular events and atherosclerosis, is positively correlated with high-risk OSA in patients with acute IS [4].

The risk of IS during sleep is heightened in patients with OSA, and treating OSA may help prevent IS during sleep [5, 6]. A systematic review has shown that improving sleep disorders can reduce the risk of recurrent stroke and death [7]. Continuous positive airway pressure (CPAP) therapy, which maintains continuous positive pressure in the airway through a respirator, has been found to improve cognitive and motor function in acute IS patients with OSA [8]. Additionally, nitrate drugs and angiotensin-converting enzyme inhibitors are independent factors that reduce the survival rate in patients with comorbid OSA and cardiovascular diseases. CPAP therapy can counteract the adverse effects of these medications by alleviating intermittent hypoxia [9].

IS and OSA share common risk factors such as obesity, hypertension, and diabetes [10, 11]. From a biological mechanism perspective, hypoxemia resulting from OSA triggers inflammation, which promotes oxidative stress and damages the vascular endothelium, leading to cerebrovascular disease and stroke. Additionally, intermittent hypoxia associated with OSA stimulates the sympathetic nervous system to release catecholamines, which mediate hypertension and further damage the vascular endothelium [12]. The progression and adverse outcomes of IS are also linked to inflammation, oxidative stress, and vascular endothelial dysfunction. Studies have shown that IS patients with OSA exhibit elevated levels of blood inflammatory markers, including tumor necrosis factor-alpha (TNF-α), interleukin-6 (IL-6), and plasminogen activator inhibitor-1 (PAI-1). Moreover, IL-6 levels are positively correlated with desaturation index and negatively correlated with oxygen saturation [13, 14], suggesting that inflammation and the immune system play a significant role in the comorbidity of OSA and IS.

Currently, IS is typically diagnosed through imaging, while OSA diagnosis requires patients to wear a multi-channel sleep monitoring device overnight, which often results in low patient compliance. This study leverages bioinformatics and machine learning techniques to identify immune-related biomarkers for IS and OSA and to develop a clinical prediction model. The model’s effectiveness was validated using an external independent dataset, offering potential new directions for the clinical prevention and diagnosis of comorbid IS and OSA.

## 2. Materials and methods

### 2.1 Data collection and processing

**Fig 1:**
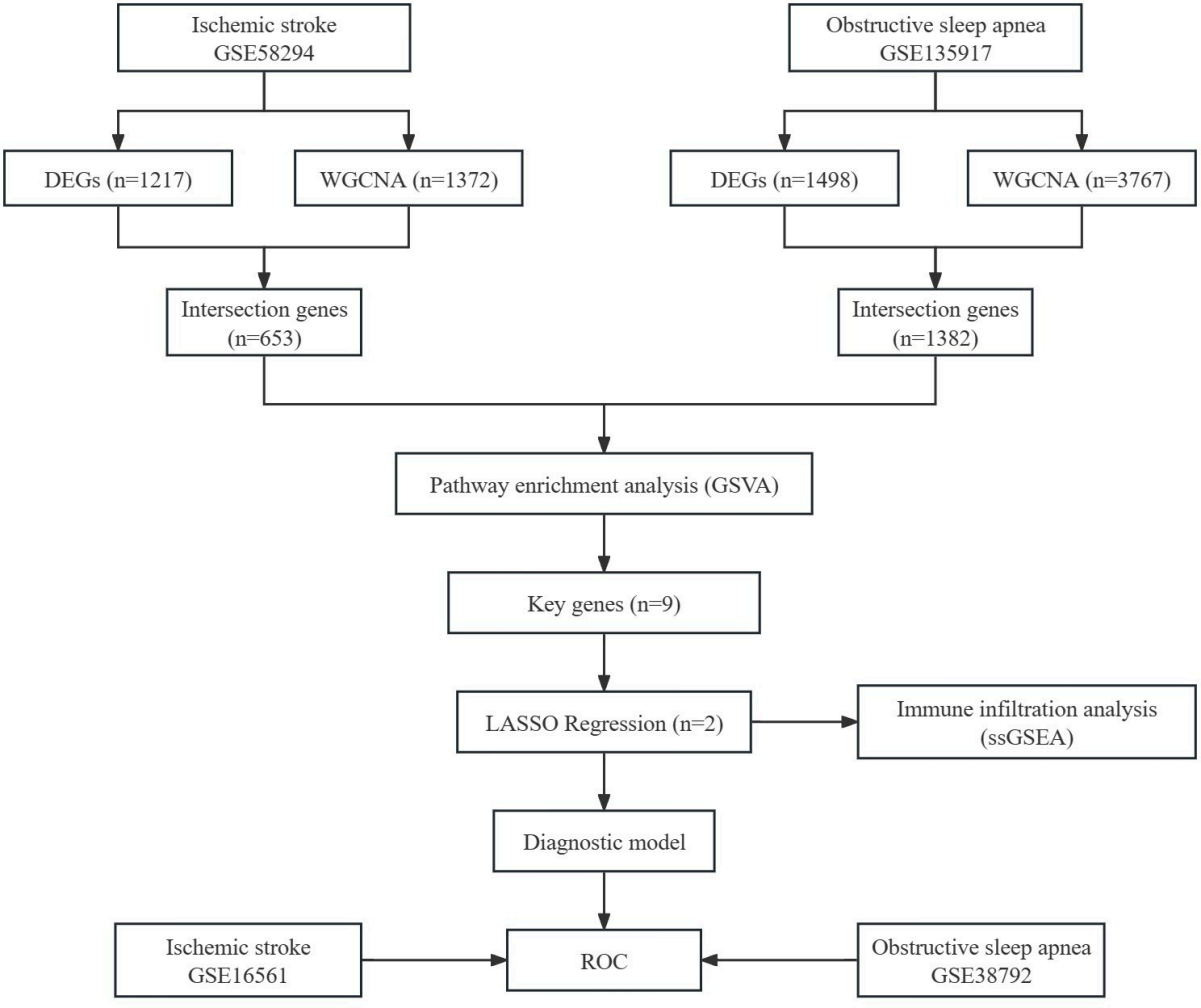
Workflow diagram. DEGs, differentially expressed genes; WGCNA, weighted gene co-expression network analysis; GSVA, gene set variation analysis; LASSO, least absolute shrinkage and selection operator; ssGSEA, single-sample gene set enrichment analysis; ROC, receiver operating characteristic.

Fig 1 illustrates the basic research workflow. All datasets were sourced from the Gene Expression Omnibus (GEO) database. The IS datasets include GSE58294 and GSE16561, while the OSA datasets include GSE135917 and GSE38792. GSE58294, based on the GPL570 platform, contains 69 ischemic stroke samples and 23 control samples. For our analysis, we selected 23 untreated IS samples and 23 control samples. GSE135917, based on the GPL6244 platform, includes 34 OSA samples, 24 OSA samples treated with CPAP, and 8 control samples. We included 34 untreated OSA samples and 8 control samples in our analysis. These datasets were used as the training set for identifying common biomarkers.

GSE16561 and GSE38792 serve as independent validation sets to assess the efficacy of the diagnostic model. GSE16561, based on the GPL6883 platform, includes 39 IS samples and 24 control samples. GSE38792, based on the GPL6244 platform, comprises 10 OSA samples and 8 control samples.

Expression matrices and annotation files for each dataset were downloaded. Probes with the same gene name were normalized using the mean, and probes without corresponding gene names were excluded. For probes with excessively large original values, log2 transformation was applied. All analyses were performed using R 4.3.2.

### 2.2 Differentially expressed genes

The Limma package was used to filter differentially expressed genes (DEGs) from the gene expression data. The threshold criteria were set at *p*.*adjust* < 0.05 and |log_2_FC| > 0.585, which corresponds to a Fold Change > 1.5.

### 2.3 Weighted gene co-expression network analysis

The WGCNA (Weighted Gene Co-expression Network Analysis) package was used to select modules with the highest disease relevance and to identify hub genes from the gene expression data. First, genes within the top 25% of variance were retained, and poor-quality data and outlier samples were excluded. Next, various power values (soft-thresholding parameters) were tested, and network fitting indices were calculated to determine the minimum power value that achieved a scale-free network topology. Subsequently, the gene expression data was partitioned into modules, each assigned a color label. The minimum module size was set to 30, and a height threshold of 0.25 was used for module merging. Hierarchical clustering was employed to construct the gene co-expression network and delineate modules, with the Topological Overlap Matrix (TOM) being retained. Finally, module eigengenes, the correlation between modules and external traits, and statistical tests for these correlations were computed.

### 2.4 Pathway enrichment analysis

Gene set information for "Homo sapiens" and the "hallmark" category was retrieved from the Molecular Signatures Database (MSigDB). Pathway enrichment analysis was performed using the GSVA package. Differential analysis was then conducted using the Limma package, with the t-value from GSVA scores serving as the metric, and an absolute value of 1 used as the threshold to differentiate upregulated and downregulated pathways. Pathways with an adjusted p-value < 0.05 were considered statistically significant.

### 2.5 Machine learning

Machine learning algorithms were used to screen core genes and develop a disease prediction model. For the IS training set, the glmnet package was employed for Least Absolute Shrinkage and Selection Operator (LASSO) regression, using 5-fold cross-validation with Mean Squared Error (MSE) as the evaluation metric. The regression coefficients corresponding to the Lambda value with the minimum cross-validation error (lambda.min) were then extracted to establish the diagnostic model.

### 2.6 Immune infiltration analysis

Immune infiltration analysis was performed using the GSVA package, specifically through single-sample gene set enrichment analysis (ssGSEA) on the gene expression data. The Spearman correlation coefficient was calculated between genes and immune cells. The gene set data for 28 immune cell types were sourced from the Tumor Immune System Interactions Database (TISIDB; S1 Table).

### 2.7 Receiver operating characteristic

The pROC package was used to plot the receiver operating characteristic (ROC) curves for the diagnostic model in both the training and validation sets. The Area Under the Curve (AUC) values were calculated to evaluate the model’s diagnostic performance, with an AUC value greater than 0.7 considered indicative of diagnostic value.

## 3. Results

### 3.1 Differentially expressed genes

**Fig 2:**
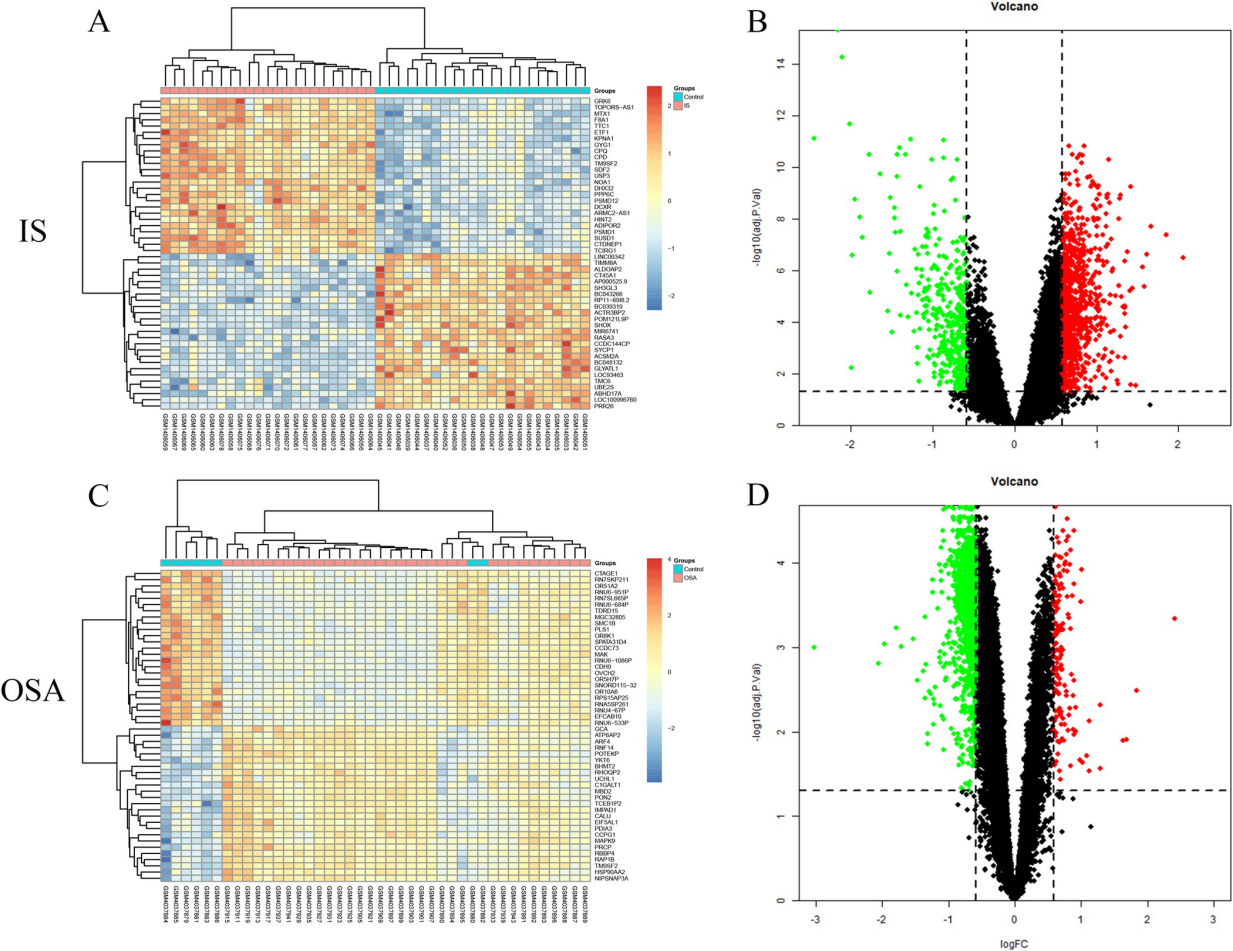
Limma differential analysis results for IS and OSA. (A, C) Heatmaps displaying the top 25 upregulated and downregulated DEGs for IS and OSA. The red module represents upregulation, and the blue module represents downregulation. (B, D) Volcano plots illustrating the DEGs for IS and OSA. Red points indicate upregulation, while green points indicate downregulation. IS, ischemic stroke; OSA, obstructive sleep apnea; DEGs, Differentially Expressed Genes.

After performing differential analysis using the Limma package, 1,217 DEGs were identified in the IS dataset, including 817 upregulated genes and 400 downregulated genes (Fig 2A and 2B). In the OSA dataset, 1,498 DEGs were screened, comprising 152 upregulated genes and 1,346 downregulated genes (Fig 2C and 2D).

### 3.2 Weighted gene co-expression network analysis

**Fig 3:**
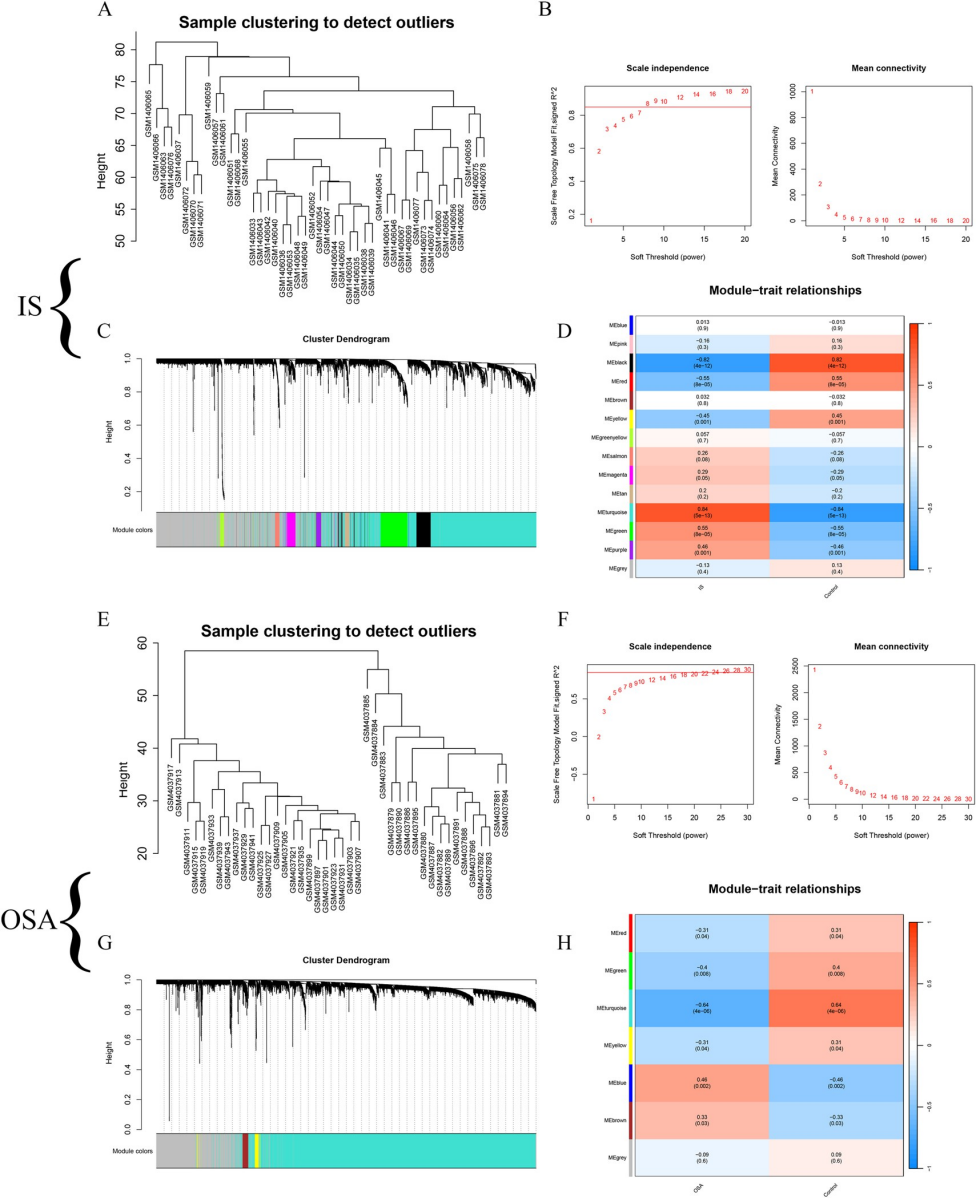
Co-expression network of IS and OSA. (A, E) Identification of outlier samples for IS and OSA. (B, F) Different soft-thresholds and corresponding network fitting metrics for IS and OSA. (C, G) Gene co-expression networks constructed through hierarchical clustering, revealing different color modules. (D, H) Correlation of modules in IS and OSA with diseases, represented by correlation coefficient values. IS, ischemic stroke; OSA, obstructive sleep apnea.

After conducting an outlier sample test, no outlier samples were found in either the IS or OSA datasets (Fig 3A and 3E). In the IS dataset, a fit index greater than 0.85 was observed, and the network achieved a scale-free topology with a soft threshold of 8 (Fig 3B). Hierarchical clustering was used to construct a gene co-expression network, resulting in 14 color modules (Fig 3C). The Turquoise module showed the strongest positive correlation (Fig 3D), leading to the identification of 1,372 hub genes. In the OSA dataset, a fit index greater than 0.85 was also observed, with the network reaching scale-free topology using a soft threshold of 18 (Fig 3F). Hierarchical clustering identified 7 color modules (Fig 3G). The Turquoise module exhibited the strongest negative correlation (Fig 3H), resulting in the identification of 3,767 hub genes.

### 3.3 Pathway enrichment analysis

**Fig 4:**
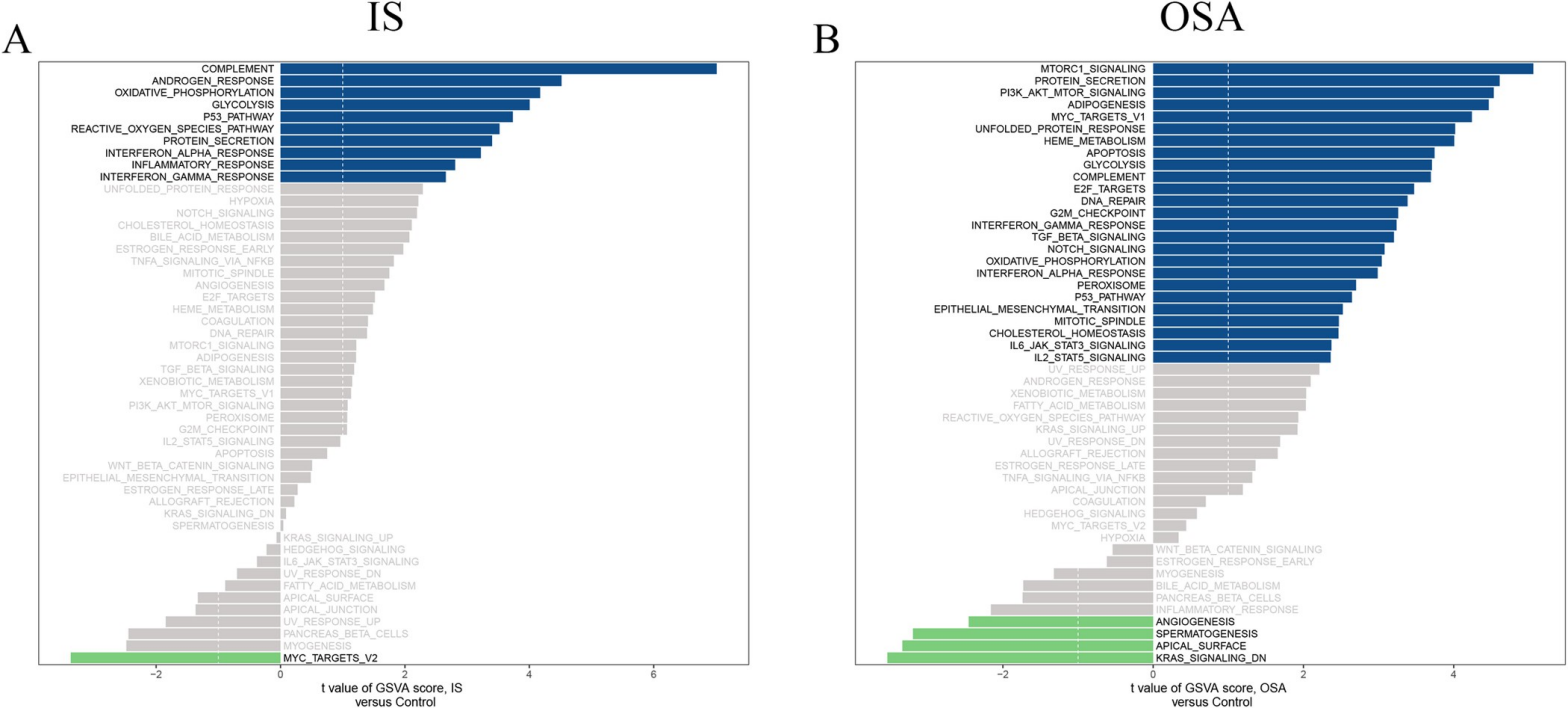
GSVA pathway scores for IS and OSA. (A, B) GSVA scores for IS and OSA, where blue represents upregulated pathways, green represents downregulated pathways, with the x-axis indicating scores and the y-axis indicating pathway names. IS, ischemic stroke; OSA, obstructive sleep apnea; GSVA, Gene Set Variation Analysis.

The GSVA results reveal 25 pathways commonly upregulated and 3 pathways commonly downregulated in both IS and OSA (Fig 4A and 4B). Limma differential analysis indicates that pathways such as complement, oxidative phosphorylation, glycolysis, the p53 pathway, protein secretion, interferon alpha response, and interferon gamma response are significantly upregulated compared to the control group (*adj*.*P*.*Val* < 0.05).

### 3.4 Machine learning

**Fig 5:**
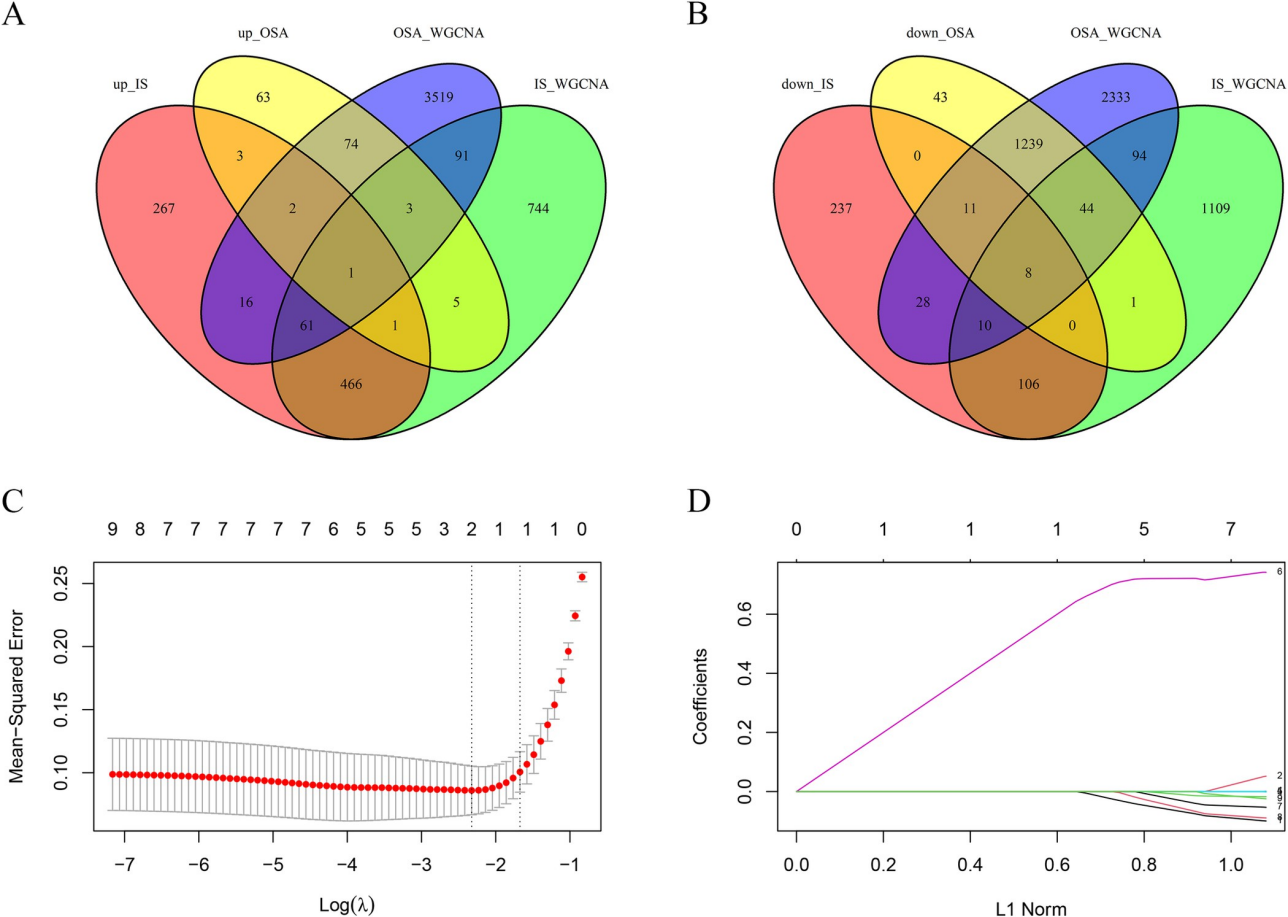
LASSO regression for screening candidate diagnostic biomarkers. (A, B) Venn diagram of the intersection between DEGs and hub genes. (C) Results of cross-validation in LASSO regression, including cross-validation error curves at different penalty levels. (D) Chart of L1 norm in LASSO regression, observing the sparsity of coefficients at different lambda values. IS, ischemic stroke; OSA, obstructive sleep apnea; DEGs, Differentially Expressed Genes; WGCNA, Weighted Gene Co-expression Network Analysis; LASSO, least absolute shrinkage and selection operator.

The intersection of DEGs between IS and OSA with key module hub genes identified 9 key genes, including 1 upregulated gene and 8 downregulated genes (Fig 5A and 5B). In the LASSO regression analysis (Fig 5C and 5D), the lambda value corresponding to the minimum cross-validation error (lambda.min) was selected. Genes with non-zero coefficients, specifically *TM9SF2* and *CCL8*, were extracted, and a diagnostic model was established based on the regression coefficients of these genes.

### 3.5 Immune infiltration analysis

**Fig 6:**
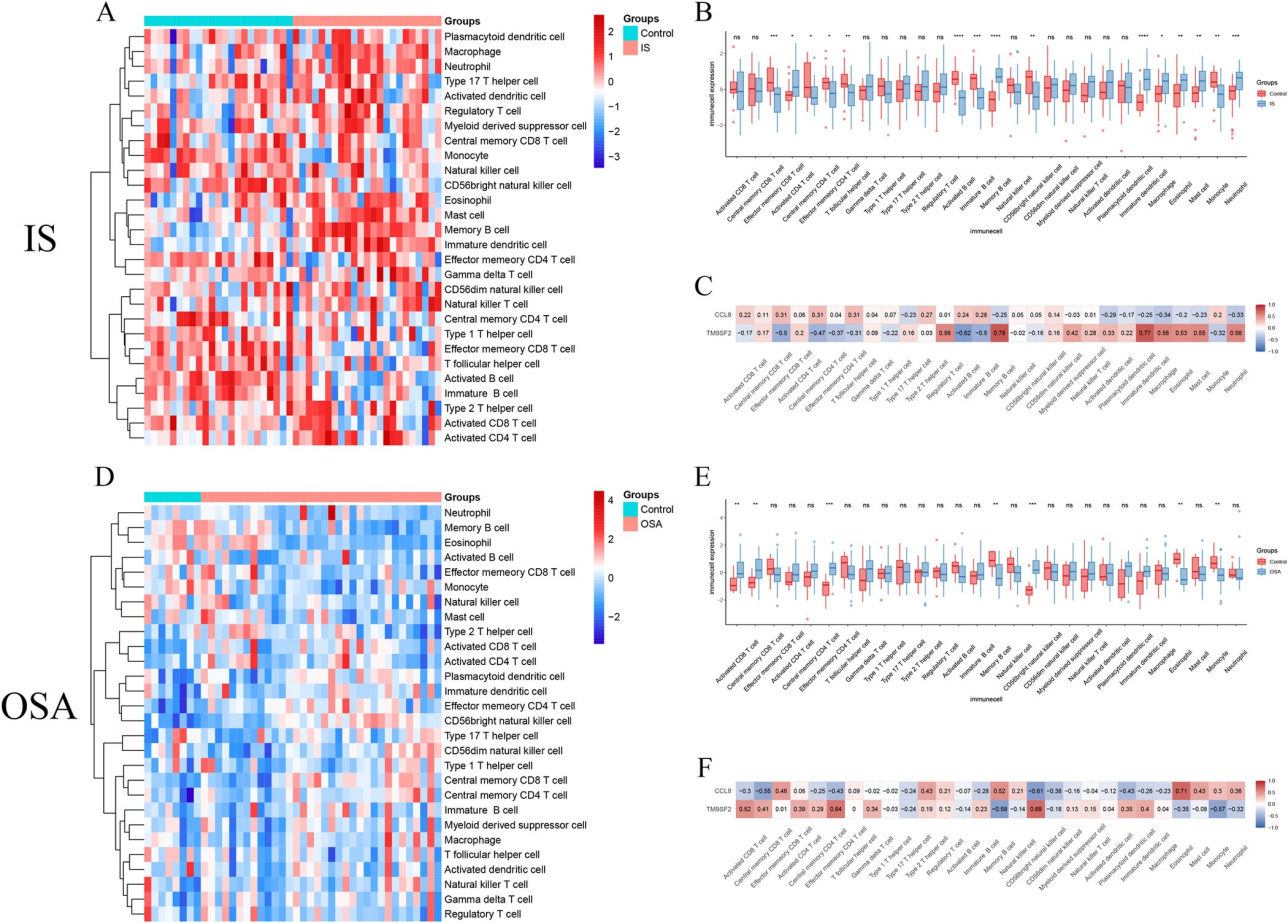
ssGSEA is employed to analyze immune infiltration in IS and OSA. (A, D) ssGSEA heatmaps for IS and OSA, where red represents upregulated immune cells and blue represents downregulated immune cells. (B, E) ssGSEA box plots for IS and OSA, with the x-axis representing 28 types of immune cells and the y-axis representing immune cell scores. Blue indicates the disease group, and red represents the control group. ns, *p* > 0.05; *, *p* < 0.05; **, *p* < 0.01; ***, *p* < 0.001. (C, F) Correlation heatmaps between core genes in IS and OSA and immune cells. Red indicates positive correlation, blue indicates negative correlation, and numbers represent correlation coefficients. IS, ischemic stroke; OSA, obstructive sleep apnea; ssGSEA, single-sample gene set enrichment analysis.

The ssGSEA heatmap illustrates the abundance of immune cells across different groups of IS and OSA samples (Fig 6A and 6D). Box plots reveal that Monocyte levels are significantly decreased in both IS and OSA samples compared to control samples (Fig 6B and 6E). Gene correlation analysis (Fig 6D and 6F) shows that in both diseases, *TM9SF2* is negatively correlated with Monocytes (Corr = -0.32/-0.57), while *CCL8* is positively correlated with Monocytes (Corr = 0.2/0.3). In IS, *TM9SF2* exhibits the strongest positive correlation with Memory B cells (Corr = 0.78), whereas *CCL8* shows the strongest negative correlation with Macrophages (Corr = -0.34). In OSA, *TM9SF2* has the strongest positive correlation with CD56bright natural killer cells (Corr = 0.68), and *CCL8* has the strongest positive correlation with Eosinophils (Corr = 0.71).

### 3.6 Receiver operating characteristic

**Fig 7:**
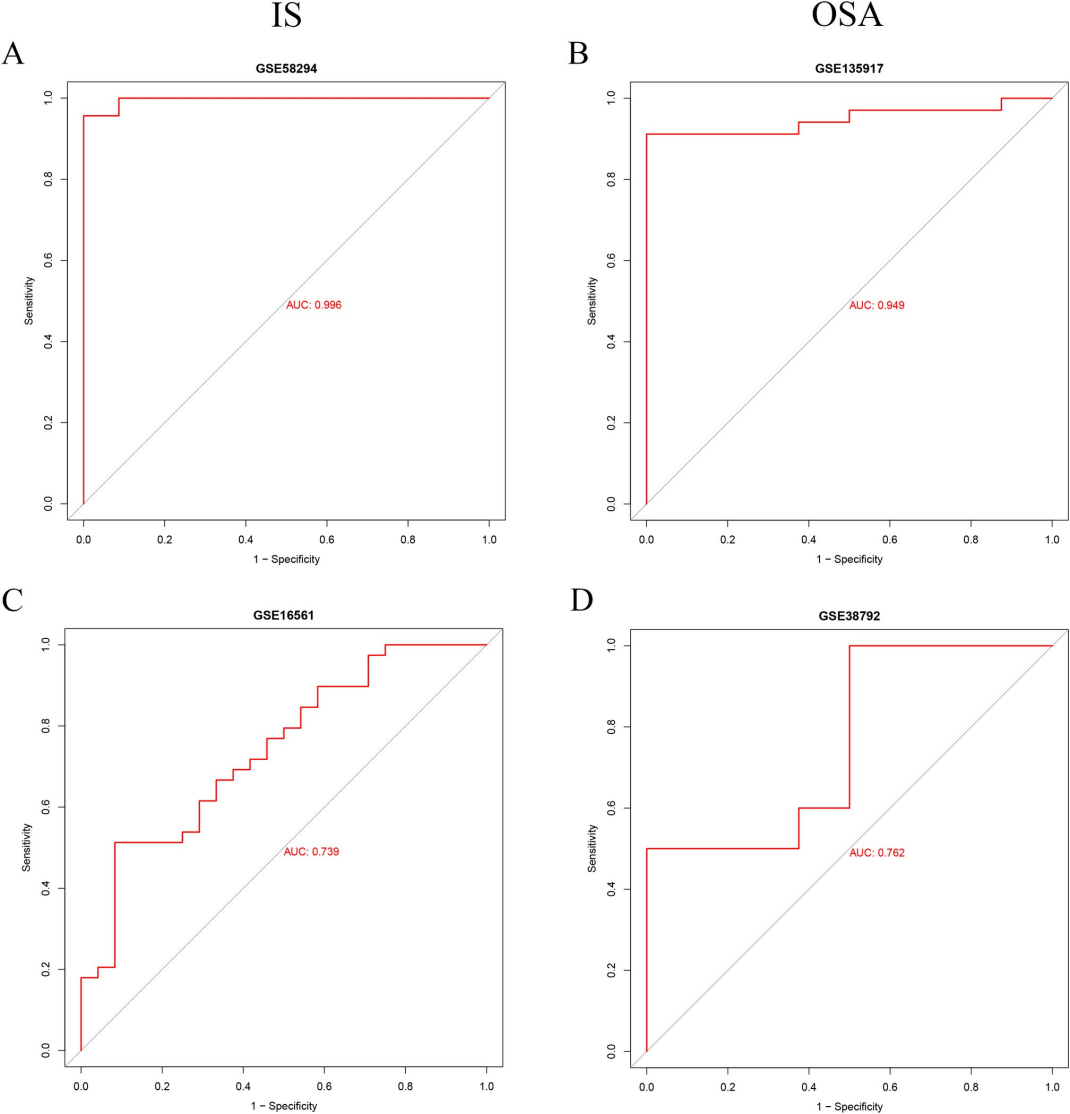
The ROC curves and AUC values of the diagnostic model. (A, B) ROC curves and AUC values of the diagnostic model in the IS and OSA training sets. (C, D) ROC curves and AUC values of the diagnostic model in the IS and OSA validation sets.

ROC curves for the diagnostic model were plotted in two training sets and two external validation sets, with AUC values calculated. The AUC values for the diagnostic model were 0.996 for GSE58294, 0.739 for GSE16561, 0.949 for GSE135917, and 0.762 for GSE38792 (Fig 7). These results demonstrate that the diagnostic model exhibits excellent accuracy in identifying the co-occurrence of IS and OSA.

## 4. Discussion

Based on microarray gene chip data for IS and OSA, two comorbid genes, *TM9SF2* and *CCL8*, were identified. Monocyte levels significantly decreased in both IS and OSA, with *TM9SF2* showing a negative correlation and *CCL8* showing a positive correlation with Monocytes. The diagnostic model, established using LASSO regression coefficients, also demonstrated excellent diagnostic value in external datasets. Extracting peripheral blood samples is more convenient and acceptable to patients compared to traditional testing methods. Preventing and early diagnosing the occurrence of IS following OSA is crucial for maintaining human health.

The GSVA results indicate that both IS and OSA involve pathways related to complement, oxidative phosphorylation, glycolysis, the p53 pathway, protein secretion, and interferon responses. (1) Complement System: The complement system is a critical component of the innate immune response. Post-IS, complement components derived from leukocytes, activated brain endothelial cells, and synthesized by glial cells and neurons are closely linked to disease progression [15]. For instance, complement components such as complement component 1q (C1q), complement component 3 (C3), and complement component 3a receptor (C3aR) play roles in neurogenesis and synaptic plasticity following IS [16]. During OSA, intermittent hypoxia induces complement-mediated release of endothelial von Willebrand factor and angiopoietin-2 [17]. Statins can restore endothelial protection against complement, mitigating downstream pro-inflammatory effects and reducing cardiovascular risk [18]. (2) Oxidative Phosphorylation: This process involves ATP synthesis through the phosphorylation of ADP within the mitochondria during aerobic respiration. Mitochondria are major targets of ischemic and hypoxic injury, and deficiencies in nutrients and oxygen can impair mitochondrial function, leading to an increased reliance on glycolysis [19]. Both IS and OSA create hypoxic environments that disrupt oxidative phosphorylation and glycolysis pathways. (3) p53 Pathway: The tumor suppressor protein p53 is crucial for regulating the cell cycle and apoptosis. In IS, Notch signaling inhibits p53 ubiquitination, which affects neural progenitor cell apoptosis and promotes disease progression [20]. Research on OSA indicates that intermittent hypoxia activates multiple p53 pathways, leading to downregulation of cell cycle proteins E2 and D1, inhibition of BV2 microglial cell proliferation, and activation of inflammation in these cells [21]. (4) Protein Secretion: The endoplasmic reticulum (ER) is essential for protein secretion, involving processes such as protein folding, glycosylation, and phosphorylation. Excessive or prolonged ER stress during IS activates apoptosis pathways that lead to neuronal death [22]. In OSA, intermittent hypoxia increases ER stress and iron death levels, contributing to myocardial damage [23]. (5) Interferon Responses: Interferons are crucial for immune regulation, including antimicrobial, antiviral, and antitumor activities, as well as inflammation. Following IS, interferon-alpha (IFN-α) signaling is activated in microglial cells via the IFN-alpha/beta receptor 1 (IFNAR1) [24]. The pro-inflammatory cytokine interferon-gamma (IFN-γ) regulates post-IS inflammation and exosome function, aiding neural stem cell repair [25, 26]. After OSA, IFN-γ primarily modulates systemic inflammation [27].

Transmembrane 9 superfamily member 2 (*TM9SF2*) is a transmembrane protein that has been identified as a novel oncogene in colorectal cancer. Approximately one-third of colorectal cancer patients show overexpression of *TM9SF2* mRNA, which may be regulated by the Ets family transcription factor ELF1 [28]. Furthermore, *TM9SF2* is positively regulated by long intergenic non-protein coding RNA 1232 (*LINC01232*), which enhances its oncogenic activity by recruiting eukaryotic translation initiation factor 4A3 (EIF4A3) to stabilize *TM9SF2* mRNA [29]. *TM9SF2* also plays a role in glycolipid regulation; its deficiency affects the localization of globotriaosylceramide (Gb3) synthase but does not impact its activity [30, 31]. A multi-omics study links *TM9SF2* to early-onset severe depression and shows a strong positive correlation with glutamine [32]. Although research on *TM9SF2* in cerebrovascular diseases and sleep disorders is currently lacking, our findings suggest it could serve as a promising diagnostic and therapeutic target in these fields.

C-C motif chemokine 8 (*CCL8*) is a member of the CC chemokine subfamily and plays a role in the migration and invasion of cancer cells in colorectal and breast cancers [33, 34]. It is involved in immune cell infiltration and inflammation in conditions such as allogeneic kidney transplantation and lipopolysaccharide-induced lung injury [35, 36]. In the context of atherosclerosis, a major risk factor for stroke, *CCL8* is crucial. It is produced by activated inflammatory and endothelial cells, mediating the progression of atherosclerosis [37]. In cerebral ischemia models, *CCL8* derived from microglial cells contributes to infarct progression by mediating CCR2/CCR5 CD8 T cell infiltration [38]. *CCL8* has also been shown to mediate neuroinflammation and contribute to neuropathic pain [39]. OSA is associated with changes in inflammatory mediators [40], and *CCL8* is likely involved in this process. Thus, *CCL8* plays a significant role in immune cell infiltration and activation of inflammatory cells, potentially affecting the progression of both IS and OSA through these mechanisms.

Immune and inflammatory responses are critical in the pathophysiological cascade of acute injury and chronic progression following IS. In IS, there is a complex interplay among monocytes, T lymphocytes, B lymphocytes, neutrophils, and microglial cells. While these responses contribute positively by removing necrotic tissue, they can also cause additional damage to healthy brain cells, leading to chronic inflammation [41]. Research indicates a positive correlation between OSA symptoms and systemic immune inflammation indices in American adults [42], underscoring the importance of inflammation and immune mechanisms in OSA. Monocytes, also known as monocyte macrophages, are crucial components of the immune system responsible for engulfing and clearing pathogens, cell debris, and foreign substances. Following cerebral vascular occlusion, microglial cells are the first to activate, followed by the differentiation of monocytes into macrophages, which collectively participate in the immune response. Macrophages can polarize into pro-inflammatory or anti-inflammatory phenotypes. The pro-inflammatory phenotype exacerbates neuronal damage in the later stages of IS [43]. Intermittent hypoxia in OSA enhances the chemotaxis and adhesion of THP-1 monocytes (a human monocytic leukemia cell line), promoting the polarization of macrophages toward a pro-inflammatory phenotype [44]. Studies have shown that compared to controls, gene expression in monocytes is generally downregulated in small vessel, large vessel, and cardioembolic strokes [45]. Additionally, OSA patients exhibit a reduction in classical monocytes in peripheral blood compared to healthy subjects, which may be closely related to changes in monocyte subpopulations [46]. This is consistent with our findings from immune infiltration analysis.

In summary, we utilized bioinformatics and machine learning algorithms to identify key biomarkers (*TM9SF2* and *CCL8*) for diagnosing IS and OSA. Our investigation also explored changes in the immune microenvironment of IS and OSA and their correlation with these core genes. A diagnostic model was developed using LASSO regression, and its high diagnostic efficacy was validated in external datasets, offering improved convenience and acceptability over traditional clinical diagnostic methods. Given the novelty of this research and the significant clinical overlap between IS and OSA, our study provides valuable insights for disease prevention and diagnosis. However, our study has certain limitations. Future research should focus on determining the protein expression levels of these genes to better understand their roles in transcription, translation, and overall biological function. Additionally, while our study highlights genetic-level changes identified through gene chip sequencing, it does not directly address or exclude the potential impact of inherited genetic factors on thrombotic susceptibility in OSA patients. These genetic changes may represent acquired alterations related to the disease rather than inherited predispositions.

## 5. Conclusion

In summary, two immune-related core genes, *TM9SF2* and *CCL8*, were identified as common to both IS and OSA. The diagnostic model developed based on these genes may be used to predict the risk of IS in OSA patients.

## Supporting information

S1 Table The immune meta gene lists for 28 immune cell types. (XLSX)
